# Identification and Pharmaceutical Characterization of a New Itraconazole Terephthalic Acid Cocrystal

**DOI:** 10.3390/pharmaceutics12080741

**Published:** 2020-08-06

**Authors:** Ricardo Machado Cruz, Tereza Boleslavská, Josef Beránek, Eszter Tieger, Brendan Twamley, Maria Jose Santos-Martinez, Ondřej Dammer, Lidia Tajber

**Affiliations:** 1 School of Pharmacy and Pharmaceutical Sciences, Trinity College Dublin, Dublin 2, Ireland; cruzr@tcd.ie (R.M.C.); santosmm@tcd.ie (M.J.S.-M.); 2 Zentiva, k.s., U Kabelovny 130, 102 37 Prague, Czech Republic; tereza.boleslavska@zentiva.com (T.B.); josef.beranek@zentiva.com (J.B.); tiegereszter@gmail.com (E.T.); ondrej.dammer@zentiva.com (O.D.); 3 Department of Chemical Engineering, University of Chemistry and Technology, Prague, Technická 5, 166 28 Prague, Czech Republic; 4 School of Medicine, Trinity College Dublin, Dublin 2, Ireland; twamleyb@tcd.ie; 5 School of Chemistry, Trinity College Dublin, Dublin 2, Ireland

**Keywords:** itraconazole, terephthalic acid, cocrystal, crystal structure, mechanochemistry, solid-state, thermal analysis, wettability, dissolution

## Abstract

The crystallization of poorly soluble drug molecules with an excipient into new solid phases called cocrystals has gained a considerable popularity in the pharmaceutical field. In this work, the cocrystal approach was explored for a very poorly water soluble antifungal active, itraconazole (ITR), which was, for the first time, successfully converted into this multicomponent solid using an aromatic coformer, terephthalic acid (TER). The new cocrystal was characterized in terms of its solid-state and structural properties, and a panel of pharmaceutical tests including wettability and dissolution were performed. Evidence of the cocrystal formation was obtained from liquid-assisted grinding, but not neat grinding. An efficient method of the ITR–TER cocrystal formation was ball milling. The stoichiometry of the ITR–TER phase was 2:1 and the structure was stabilized by H-bonds. When comparing ITR–TER with other cocrystals, the intrinsic dissolution rates and powder dissolution profiles correlated with the aqueous solubility of the coformers. The rank order of the dissolution rates of the active pharmaceutical ingredient (API) from the cocrystals was ITR–oxalic acid > ITR–succinic acid > ITR–TER. Additionally, the ITR–TER cocrystal was stable in aqueous conditions and did not transform to the parent drug. In summary, this work presents another cocrystal of ITR that might be of use in pharmaceutical formulations.

## 1. Introduction

A pharmaceutical cocrystal can be defined as a multicomponent crystal wherein at least one component is the active pharmaceutical ingredient (API) and is in a well-defined stoichiometric ratio and bonded by non-covalent interactions with the other component(s), i.e., the coformer(s) [1,2]. These interactions are mainly intermolecular hydrogen bonds between functional groups forming supramolecular synthons, and moreover, additional weaker interactions such as van der Waals forces, π-stacking or halogen bonds may also help to stabilize the cocrystal structure [3,4]. The purpose of synthesizing pharmaceutical cocrystals is to improve the characteristics of APIs, such as the aqueous solubility, dissolution rates and stability [5].

Itraconazole (ITR) is an API with a broad-spectrum antifungal activity used for the treatment of topical and systemic mycoses as well as for prophylaxis in immunosuppressed patients [6,7]. ITR, due to a low aqueous solubility but a high systemic absorption, is considered a class II drug according to the biopharmaceutics classification system (BCS) [8]. Indeed, ITR has a very low solubility in water (around 1 ng/mL), which increases in an acidic medium (4 µg/mL) [9], thus the poor solubility is the limiting factor for ITR absorption. To improve the poor physicochemical properties, ITR can be converted into disordered forms such as liquid crystalline [10,11,12] and amorphous structures, as well as polymer-based solid dispersions [12]. The commercial solid dosage formulation of ITR, Sporanox^®^, is available as pellets enclosed in oral capsules [13]. The pellets are manufactured by spray-layering a solution of ITR and hydroxypropyl methylcellulose (HPMC) on sucrose beads. Then, the drug-coated beads are treated with a seal coating polymer layer (polyethylene glycol (PEG) 20,000 Da) to prevent the sticking of the beads. The API in these pellets is in the amorphous state [13]. Since the physical stability of drug in an amorphous phase can be short-lived, a crystalline, but a better soluble form of ITR would be preferred.

The development of ITR cocrystals was first achieved combining this API with aliphatic dicarboxylic acids used as coformers, including fumaric acid, succinic acid, l-malic acid, l-tartaric acid, d-tartaric acid and d,l-tartaric acid [14]. Remenar’s work reported a remarkable improvement of the ITR dissolution with an approximately 20-fold enhancement of the cocrystals prepared with l-malic and l-tartaric acids, which had dissolution profiles comparable to the commercial form of ITR, Sporanox^®^ capsules. In another work, Shevchenko et al. [15] produced ITR cocrystals with coformers of different carbon chain lengths and determined that linear aliphatic dicarboxylic acids should have no more than seven carbon atoms in their chains to successfully form a cocrystal with ITR. That work also demonstrated that cocrystallisation is an efficient approach to improve the dissolution rates of ITR. However, a recent study presented that an ITR cocrystal with an aliphatic dicarboxylic acid comprising more than seven carbon atoms, suberic acid, can be prepared by rapid solvent evaporation and spray drying, resulting in the material being able to dissolve rapidly. This cocrystal had a 39 times faster intrinsic dissolution rate than the crystalline ITR [16].

**Figure 1:**
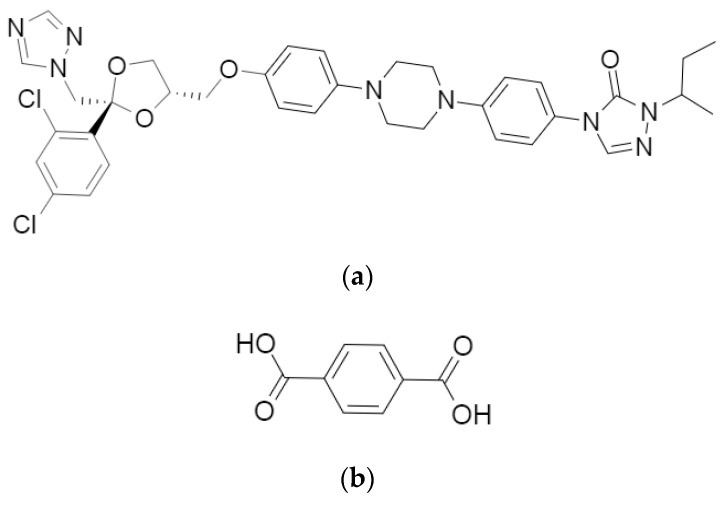
Molecular structures of (**a**) itraconazole (ITR) and (**b**) terephthalic acid (TER).

Little is published on the structural analysis of ITR cocrystals, nevertheless, such studies on the ITR and succinic acid (SUC) cocrystal revealed that this form has a trimeric building block, where two molecules of the API are oriented antiparallel to one another and bridged by the coformer. The main interactions found to be responsible for the intermolecular arrangement of this structure were H-bonds formed by the hydroxyl moiety located on both sides of the acid and one of the nitrogen atoms of the 1,2,4-triazole ring of each ITR molecule (Figure 1) [14].

Terephthalic acid (TER) is a benzenedicarboxylic acid with the carboxylic groups attached in positions one and four (Figure 1). TER has very low toxicity and has been employed in the cocrystallization of a few APIs, such as apovincamine [17], betulin [18], isoniazid [19] and gabapentin [20]. Considering that most investigations on the identification of ITR cocrystals are limited to aliphatic dicarboxylic acids [14,15,16,21], coformers with other architecture appears to be unexplored. Therefore, this work carried out an experimental screening aiming to identify a possible new cocrystal of ITR combining this API with TER to ascertain the relevant molecular elements in the coformers that enable the formation of new cocrystal phases of ITR. This new cocrystalline form was extensively evaluated regarding its solid-state characteristics, as was the impact ITR cocrystallisation had on a range of pharmaceutical properties. Finally, the cocrystal dissolution was studied including an intrinsic dissolution study, a simple mixture with lactose as well as a mixture comprising excipients used in the commercial ITR formulation, comparing the performance of the new cocrystal with those based on aliphatic dicarboxylic acids.

## 2. Materials and Methods

### 2.1. Materials

Itraconazole (ITR) was purchased from Glentham Life Sciences Ltd. (Whitshire, UK). MeOH (HPLC grade) and terephthalic acid (TER) were purchased from Sigma-Aldrich (Arklow, Ireland). All other ingredients, such as solvents, solution and buffer components, as well as polymers, were kindly provided by Zentiva (Prague, Czech Republic).

### 2.2. Methods

#### 2.2.1. Neat Grinding (NG) and Liquid-Assisted Grinding (LG)

For these preparations, 40 mg of ITR and an amount of TER corresponding to 2:1, 1:1 and 1:2 of API-coformer molar ratios were carefully weighted. Afterwards, the compounds were ground for 30 s using an agate mortar and pestle.

For liquid-assisted grinding, two drops of methanol were added to the mixture of powders.

#### 2.2.2. Cocrystallisation by Slurrying

In this method, solutions of ITR at 0.74 and 2.20 mg/mL were prepared in methanol (MeOH) and acetone, respectively. Then, a 1 mL aliquot of each solution was transferred to a 1.5 mL glass vial and a quantity of TER was added in a 1:50 API-coformer molar ratio. Then, the hermetically closed vials containing the suspensions were mixed at 50 °C for 8 h and thereafter for next 4 days at room temperature using an IKA RT 15 magnetic stirrer (Germany). Afterwards, the slurries were dried at 30 °C and 100 mbar in a 3608-6CE vacuum oven (ThermoFisher Scientific™, Waltham, MA, USA).

#### 2.2.3. Cocrystallisation by Ball Milling (BM)

A quantity of 300 mg of ITR was weighted and added to an amount of each coformer (oxalic acid, succinic acid and TER) corresponding to a 2:1 API-coformer molar ratio. Then, the powders were transferred to a 25 mL stainless-steel grind jar containing two 15 mm stainless-steel balls. Before grinding, two drops of acetone were added to the powders. The mixtures were ground in two cycles of 10 min at 25 Hz using a Retsch Mixer Mill MM 200 (Haan, Germany).

#### 2.2.4. Cocrystallisation by Slow Evaporation

A solution of ITR and TER was prepared by weighing 5.96 mg of the API and 0.70 mg of the coformer to prepare a 2:1 mole/mole mixture of the components. The powders were transferred into a glass vial and solubilized in 10 mL of methanol by sonication in a U300 H ultrasonic bath (Ultrawave, Rumney, UK) to obtain a clear solution. Then, the solution was filtered using a 0.45 µm PTFE syringe filter (Fisher Scientific, Loughborough, UK). Parafilm was used to cover the top of the vial and small holes were pierced to allow for solvent evaporation. The solution was left at room conditions until crystallization occurred.

#### 2.2.5. Freeze Drying of Itraconazole (ITR)

Firstly, a 10 mg/mL solution of ITR in dioxane was prepared by weighing 1 g of the API and solubilizing it in 100 mL of the solvent. The solution was divided into three round-bottomed flasks and frozen using liquid nitrogen while rotating using a Rotavapor R-205 (Büchi, Flawil, Switzerland). The samples were dried for 18 h using a freeze drier, ALPHA 2-4 LSC (Martin Christ, Osterode am Harz, Germany), with manifolds for a connection of NS 29/32 flasks under a vacuum of 2 × 10^−3^ mbar, between 5 and 6 m^3^/h suction and ice condenser adjusted to −85 °C. No secondary drying step was applied.

#### 2.2.6. Differential Scanning Calorimetry (DSC)

A thermal analysis was performed by carefully weighting dried samples and placing them in 40 μL aluminum pans that were sealed with a lid containing three vent holes. The samples were subjected to DSC runs in a temperature ranging from 25 to 400 °C with a heating rate of 10 °C/min using a Mettler Toledo DSC 822 e/700 (Greifensee, Switzerland) under nitrogen purge [22]. An empty aluminum pan was used as a reference. The equipment was calibrated with an indium standard.

#### 2.2.7. Powder X-ray Diffraction (PXRD)

PXRD patterns were obtained with a laboratory X’PERT PRO MPD (PANalytical, Almelo, Netherlands) diffractometer with CuKα (λ= 1.542 Å) radiation. The generator was operated at an excitation voltage of 45 kV and anodic current of 40 mA. The following scan parameters were utilized: scan type—gonio, measurement range of 2–40° 2θ, step size of 0.02° 2θ and the time per step was 200 s. The samples were placed on a zero-background silica sample holder. Alternatively, PXRD measurements were performed using a Rigaku Miniflex II, desktop X-ray diffractometer (Tokyo, Japan) equipped with a CuKα (λ = 1.54 Å) radiation X-ray source. Dried samples were mounted on a low-background silicon sample holder and scanned over a 2θ range of 2–40 degrees [23].

#### 2.2.8. Single Crystal X-ray Analysis

A monocrystal of ITR–TER with approximate dimensions of 0.030 mm × 0.140 mm × 0.150 mm was used for the X-ray crystallographic analysis. The X-ray intensity data were measured at 100 ± 2 K on a Bruker Apex Kappa Duo (Billerica, MA, USA) with an Oxford Cobra Cryosystem low-temperature device (Oxford, UK) using a MiTeGen micromount (Ithaca, NY, USA). Bruker APEX software was used to correct for Lorentz and polarization effects. The crystallographic data were analyzed using Mercury 2020.1 and CrystalExplorer (ver. 17.5) [24] software.

#### 2.2.9. Fourier-Transform Infrared Spectroscopy (FTIR) and Raman Spectroscopy

The dried powders were subjected to FT–IR spectroscopy on a PerkinElmer Spectrum 100 (Waltham, MA, USA), equipped with a universal attenuated total reflection (ATR) device and a ZnSe crystal. The FT–IR spectra of the samples were recorded in a wavelength range from 500 to 4000 cm^−1^. The spectra were acquired by averaging 10 scans taken with a resolution of 4 cm^−1^.

The Raman spectra of powders were measured directly in glass vials using a Raman Spectrometer RFS 100/S (Bruker, Billerica, MA, USA). The spectra were acquired by averaging 64 scans taken with a resolution of 4 cm^−1^ and laser power of 250 mW.

#### 2.2.10. Morphological Analysis

A Zeiss Supra variable Pressure Field Emission Scanning Electron Microscope (SEM, Ulm, Germany) equipped with a secondary electron detector and an accelerating voltage of 5 kV was used for the morphological examination. The produced powder samples were placed on carbon tabs attached to aluminum stubs and sputter coated with gold/palladium under vacuum before analysis [10].

#### 2.2.11. Contact Angle Measurements

Powdered samples were compacted into 4.5 cm in diameter disks and placed on the lifting table of a Drop Shape Analyzer DSA 25 (Krüss, Hamburg, Germany). Then, an automated dosing syringe containing water at 20 °C was used to deposit a single drop of 14 µL on the surface of the disks. The images of the water drop on the surface of the disks were recorded for 10 min by a high-resolution camera and processed by ADVANCE software ver. 1.9 (Krüss) to calculate the contact angle. Each sample had the contact angle measured in duplicate.

#### 2.2.12. Intrinsic Dissolution Rate (IDR) Study

Disks 8 mm in a diameter were prepared by compressing 50 ± 2 mg of powder in a stainless-steel cylindrical die system at approximately 100 kg/cm^2^ for 120 s. Then, the opposite side of the steel die was sealed using a rubber plug, leaving a 0.503 cm^2^ surface of the disk exposed. The stainless-steel dies were used as the disk holders and were loaded automatically by the robotic arm of the Pion inForm (Pion, Forest Row, UK) and immersed in the dissolution media. For each test, 40 mL of the medium (acetate-phosphate buffer comprising 150 mM NaCl) was preheated to 37 °C and the pH was adjusted with 0.5 M HCl to 1.2. The agitation was set to 100 rpm. A spectral scan (190–720 nm) was collected every 30 s and the concentration was calculated against the calibration curve obtained previously under identical conditions.

#### 2.2.13. Dissolution Analysis

##### 2.2.13.1. Powder Dissolution of ITR Systems Mixed with Lactose

For this procedure, samples were prepared by weighing an amount of ITR (starting material), freeze dried ITR (FD ITR), ITR–TER, itraconazole-oxalic acid cocrystals (ITR–OXA) and itraconazole-succinic acid cocrystals (ITR–SUC) containing an equivalent of 100 mg of ITR and mixing with lactose monohydrate in a 1:6 *w*/*w* API:excipient ratio to allow wettability and dispersibility in the liquid medium. Sporanox^®^ was used as the reference formulation and the pellets were removed from the capsule before the use in the dissolution experiments. The dissolution analysis was performed using a solution prepared by mixing 33 mM NaCl with 67 mM HCl (artificial gastric juice (AGJ)) containing 0.05% (*v*/*v*) of Tween 20. The pH of this mixture was then adjusted to 1.2 with 0.5 M HCl. The experiments were carried out using a standard USP II dissolution apparatus (Sotax, Aesch, Switzerland) attached to a UV-Vis spectrophotometer Specord 200 Plus (Analytik Jena, Jena, Germany). The powders were added directly to the media (900 mL) kept at 37 °C and agitated at 75 rpm for the first 45 min and at 150 rpm for the final 15 min. The aliquots were automatically taken at predefined time points (2, 5, 10, 15, 20, 25, 30, 45, 50 and 60 min) and the ITR concentration was assessed by measuring the absorbance at 255 nm.

##### 2.2.13.2. Powder Dissolution of ITR Systems Mixed with Other Excipients

A second dissolution test evaluated the dissolution of the API when physically mixed with the same excipients as those present in Sporanox^®^ (Jassen, Beerse, Belgium). For this purpose, 350 mg of ITR (starting material) and FD ITR or the amount of the ITR–TER, ITR–OXA and ITR–SUC cocrystals containing the equivalent of 350 mg of the API were carefully weighted and mixed with the other excipients in the concentrations listed in Table 1 for 5 min in 200 mL plastic bottles using a Turbula^®^ mixer (WAB group, Muttenz, Switzerland).

The dissolution analysis was carried out as described in Section 2.2.13.1, using 900 mL peak vessels in a dissolution apparatus 708-DS (Agilent, Lexington, MA, USA) coupled to an Agilent UV-Vis spectrophotometer Cary 60.

#### 2.2.14. Statistical Analysis

The statistical analysis of the data was carried out using GraphPad Prism^®^ for Windows, version 5.01, applying a Student’s *t*-test or one-way ANOVA with Tukey’s post-test with 95% of confidence when appropriate. Statistical significance was when *p* < 0.05.

## 3. Results and Discussion

### 3.1. Characterization of ITR and Terephthalic Acid (TER) Mixtures Following Neat and Liquid-Assisted Grinding

**Figure 2:**
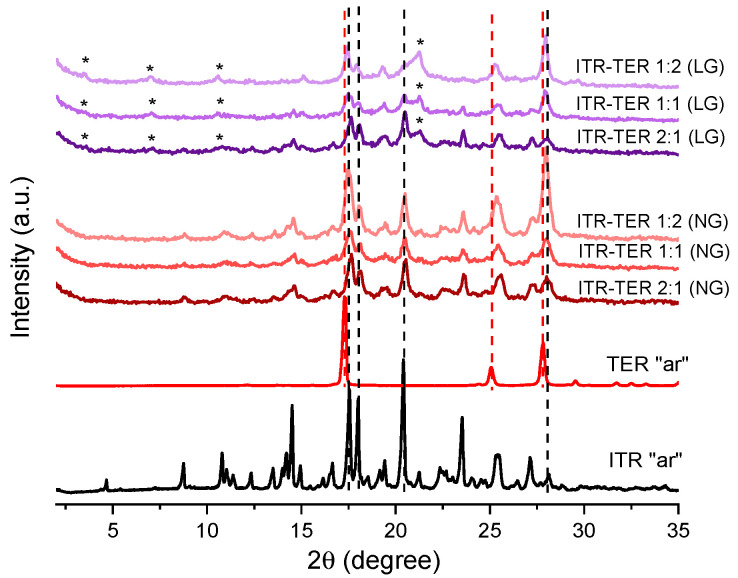
Powder X-ray diffraction (PXRD) of the ITR and TER starting material as the received powders (“ar”) and binary ITR and TER mixtures following neat grinding (NG) and liquid-assisted grinding (LG). * Indicates the distinct peak of the new phase (cocrystal), broken black lines show the position of the ITR diffraction peaks, while the broken red lines show the position of the TER diffraction peaks.

Mechanochemical methods of cocrystal production have gained a considerable interest in recent times [25,26]. Therefore, as the first approach, a neat, solvent-free (NG) grinding of ITR and TER in a few stochiometric ratios, followed by the liquid-assisted grinding (LG) was performed. The ITR “as received” material was identified as form I of itraconazole [27]. The PXRD analysis of the samples prepared by neat grinding (NG (Figure 2)) revealed that the mixtures post-processing had similar diffractograms to those of ITR with two Bragg peaks at 17.5 and 17.95° 2θ, corresponding to TER. The samples prepared by LG had additional, but weak, Bragg peaks at 3.5, 7.0 and 21.2° 2θ, which were absent in the parent materials, indicating the formation of a new phase. Interestingly, the TER component in all samples post NG and LG appeared to be at least partially amorphous, as evidenced by the weak or absent diffraction peaks of TER. Therefore, the LG method is more efficient in producing the ITR–TER cocrystal than NG most likely because the cocrystallisation process by LG was facilitated by the presence of methanol. Indeed, the use of solvents in cocrystallisation screening is common and the impact of the solvent is described as catalytic, since it is used in a very small quantity and is also not part of the final cocrystal [28].

**Figure 3:**
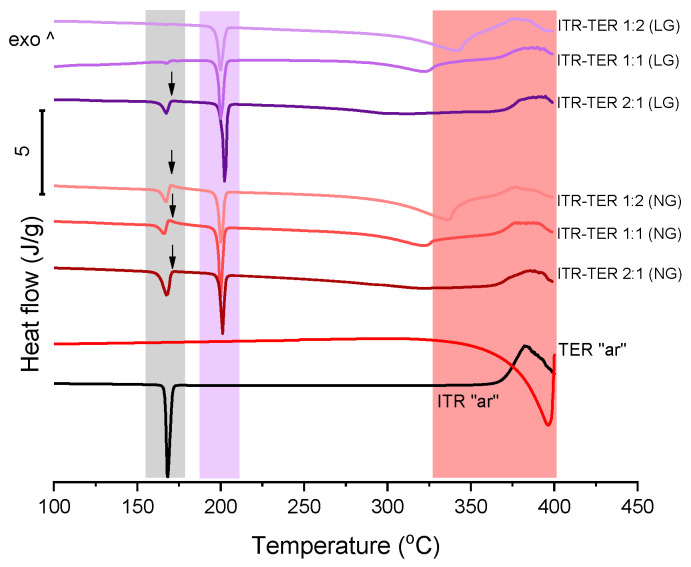
Differential scanning calorimetry (DSC) of the ITR and TER starting material, as received powders (“ar”) and binary ITR and TER mixtures following neat grinding (NG) and liquid-assisted grinding (LG). The area highlighted in grey indicates the ITR melting region, in purple the cocrystal melting range and in light red the TER melting and degradation. Arrows indicate the presence of an exothermic peak immediately following the ITR melting event.

A DSC analysis of ITR and TER on their own showed that the drug melted at around 166 °C [12], while TER melted with sublimation at around 350 °C [29] (Figure 3). A thermal analysis of the binary ITR–TER mixtures post-processing showed thermograms with a sharp endothermic peak at 198 °C (Figure 3), which was absent in the parent compounds and indicated the melting of the new phase. However, all these mixtures also had an endothermic peak with an onset at 164 °C (Figure 3), assigned to the melting of ITR. This peak in the ITR–TER 1:1 (LG) and ITR–TER 1:2 (LG) systems was almost imperceptible, with enthalpies of 2.6 and 0.7 J/g, respectively. In contrast, the ITR peak in the samples ITR–TER 1-1 (NG) and ITR–TER 1-2 (NG) had an enthalpy of 13.5 and 18.4 J/g, respectively, indicating that the methanol used in LG contributed to a greater conversion of ITR into the new phase. A small exothermic peak was detected for all NG samples and ITR–TER 1:2 (LG) immediately followed the ITR peak, consistent with the previous studies on the thermal behavior of binary mixtures where the formation of a cocrystal was observed upon heating [30]. Thus, in relation to NG mixtures, the cocrystal melting peak, visible in DSC traces, is of the new form which appears upon heating. Additionally, what can be concluded from the DSC study is that a broad endothermic peak with an onset at around 310 °C for the ITR–TER 1:1 samples and an onset at around 320 °C for the ITR–TER 1:2 systems was detected, assigned to the excess of TER. It may suggest that the potential stoichiometry of the ITR–TER cocrystal might be 2:1.

### 3.2. Properties of ITR and TER Samples Made by Slurring, Evaporation and Ball Milling Methods

**Figure 4:**
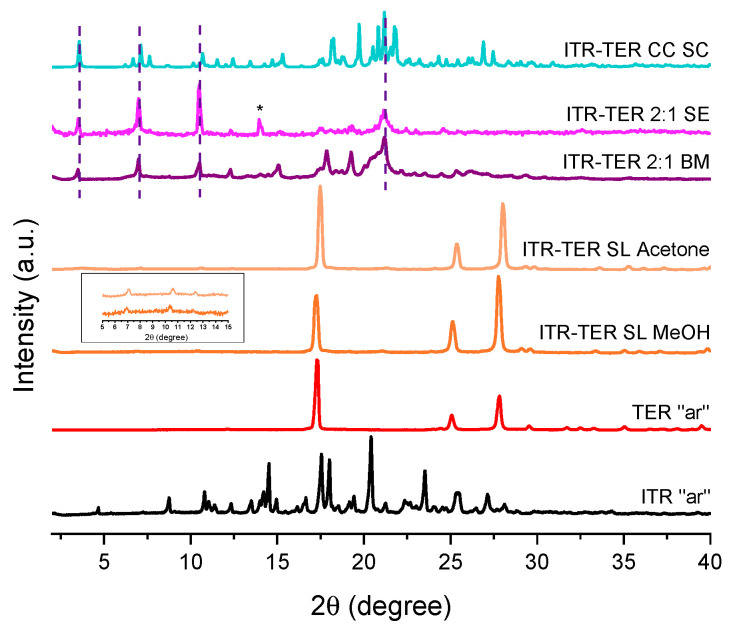
PXRD patterns of the ITR and TER starting material, as received powders (“ar”), samples produced by the slurry method from methanol (ITR–TER SL MeOH) and acetone (ITR–TER SL acetone), the ball milled sample containing ITR and TER in a 2:1 stoichiometric ratio (ITR–TER 2:1 BM), the sample obtained by the slow evaporation of ITR and TER in a 2:1 stoichiometric ratio from methanol (ITR–TER 2:1 SE) and a powder diffraction pattern of the ITR–TER cocrystal calculated from the single crystal X-ray data (ITR–TER CC SC). * Indicates the peak of ITR, broken lines indicate the position of key diffraction peaks of the cocrystal, while the insert shows the presence of the cocrystal peaks in the samples obtained by the slurry method.

A PXRD analysis of samples obtained by the slurring of ITR and TER in methanol and acetone was very similar to that of TER and indicated an incomplete conversion to the cocrystal, with only weak intensity Bragg peaks of the new phase visible at approximately 7.0, 10.4, 12.4° 2θ (Figure 4). A more successful method was the slow evaporation of ITR and TER from methanol, and the diffraction pattern of the 2:1 ITR–TER system is presented in Figure 4. A number of diffraction peaks characteristic of the cocrystal phase can be discerned, however the peak at 15.0° 2θ can be described as the ITR starting material powder. The method that gave the purest cocrystal, based on the PXRD results, was ball milling (Figure 4). The ITR–TER 2:1 system had a diffraction pattern distinct from those of the starting material powders, particularly due to the peaks at 3.5, 7.0, 10.5, 12.4, 17.8, 19.3 and 21.2° 2θ. Therefore, ball milling, as the most efficient method of cocrystal production, was used to prepare the cocrystal for further characterizations.

**Figure 5:**
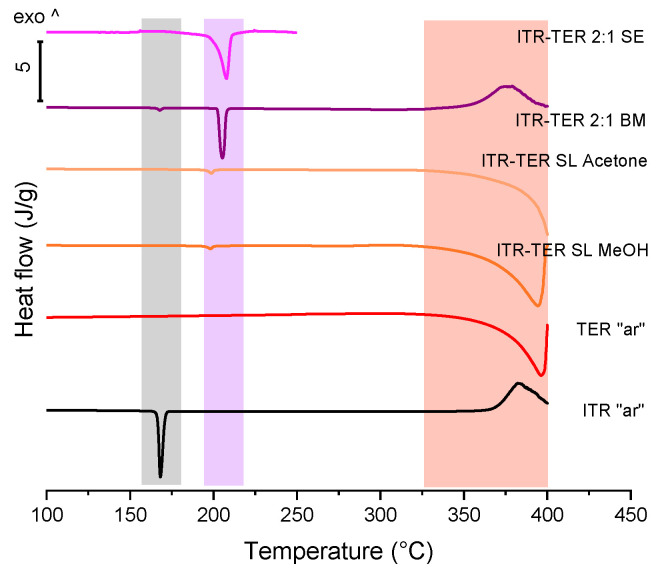
DSC thermograms of the ITR and TER starting material, as received powders (“ar”), samples produced by the slurry method from methanol (ITR–TER SL MeOH) and acetone (ITR–TER SL acetone), the ball milled sample containing ITR and TER in the 2:1 stoichiometric ratio (ITR–TER 2:1 BM) and the sample obtained by slow evaporation of ITR and TER in the 2:1 stoichiometric ratio from methanol (ITR–TER 2:1 SE). The area highlighted in grey indicates the ITR melting region, in purple the cocrystal melting range and in light red the TER melting and degradation.

A DSC analysis (Figure 5) confirmed the above XPRD data. The samples prepared by the slurring of ITR and TER in methanol and acetone had low magnitude peaks with an onset at 195 °C, which corresponded to the melting of the cocrystal phase. In both samples this peak had the normalized enthalpy of transition of 8.1 J/g. DSC also confirmed that these samples were mainly composed of TER. The sample obtained by solvent evaporation displayed a characteristic event of cocrystal melting, although the peak was broader than that of the ball milled sample. The thermal analysis of the ITR–TER 2:1 ball milled system (Figure 5) verified that ball milling was more efficient in producing the cocrystal, with a sharp melting peak with a normalized enthalpy of 83.7 J/g.

### 3.3. Infrared and Raman Spectroscopy

**Figure 6:**
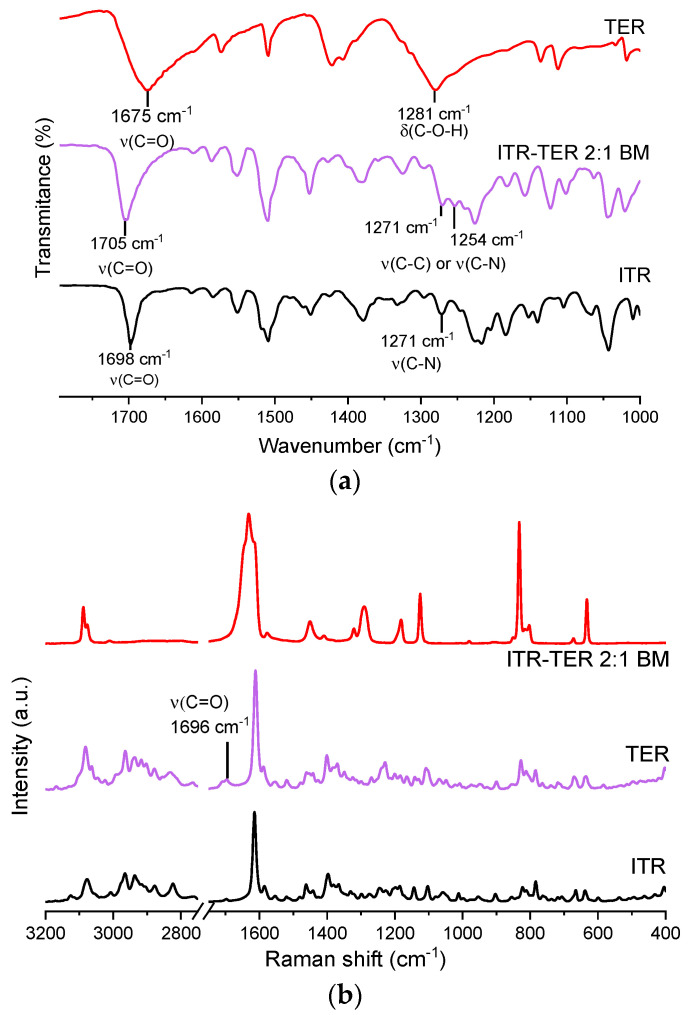
(**a**) Fourier-transform infrared spectroscopy (FT-IR) spectra of the ITR and TER starting material powders as well as the ITR–TER cocrystal prepared by ball milling (ITR–TER 2:1 BM); (**b**) Raman spectra of the ITR and TER starting material powders as well as the ITR–TER cocrystal prepared by ball milling (ITR–TER 2:1 BM). ν–stretching.

The IR spectrum of the ITR–TER cocrystal showed an intense band at 1705 cm^−1^, which was also present in the ITR and TER spectra, at 1698 cm^−1^ and 1675 cm^−1^, respectively (Figure 6a). Strong peaks in this range are normally caused by the stretching of C=O [31]. As this peak appears at a higher wavenumber in the cocrystal than in the parent compounds, this suggests that the new H-bond in the cocrystal might be weaker than the one in TER, as ITR does not form any intermolecular H-bonds. Another area of interest was the fingerprint region, between 1200 and 1300 cm^−1^. In the TER spectrum, a broad strong peak at 1281 cm^−1^, most likely from the –C–O–H vibration, was seen, while in ITR, a peak at 1271 cm^−1^ was assigned to aromatic C–N stretching [32,33]. In the cocrystal, a number of medium intensity bands were detected around this area.

Raman spectroscopy confirmed the presence of the new phase and the spectrum of the cocrystal (Figure 6b) was significantly different when compared to those of ITR and TER, which indicate interactions between the API and the coformer forming supramolecular interactions [34]. In the pure coformer, H-bonded dimers are formed by the –COOH groups, with the C=O moiety acting as the H-bond acceptor and the –OH moiety being the H-bond donor. In the cocrystal, the acid dimer arrangement is disrupted, which results in a shift of the peak assigned to the C=O stretching band to 1696 cm^−1^ in the cocrystal spectrum, while in the conformer, the band is located at 1633 cm^−1^. This effect was also observed by Shevchenko et al. [15], who investigated the formation of ITR cocrystals with aliphatic dicarboxylic acid of varying chain lengths, including succinic acid, malonic acid, and oxalic acid. In that work, the authors detected a peak shift of the C=O vibration in all produced cocrystals.

### 3.4. Crystallographic Analysis

**Figure 7:**
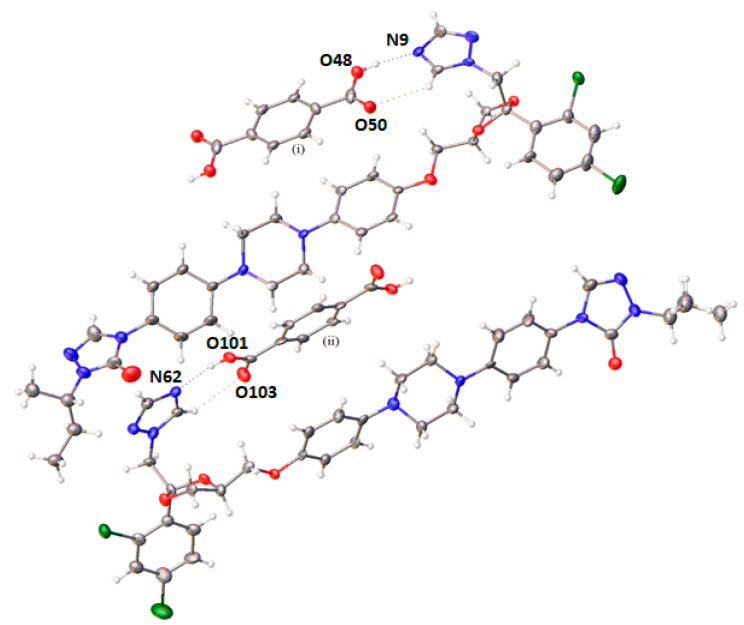
Crystal structure of the ITR–TER cocrystal (symmetry transformations used to generate equivalent atoms: (i) −x, 2-y, 1-z; (ii) 1-x, 1-y, 1-z) showing the major disorder in the ITR moiety only.

Single crystal X-ray diffraction (SC-XRD) showed that the cocrystal had a triclinic unit cell composed of four molecules (Z = 4) in a P$\bar{1}$ space group (Table S1). The asymmetric unit crystal was formed by two ITR molecules and two halves of a TER molecule. The stoichiometry of the cocrystal was determined as 2 moles of ITR per 1 mole of TER. In this arrangement, the ITR molecules are in an antiparallel orientation with a TER molecule, which is sandwiched in the formed space by the drug molecules (Figure 7). SC-XRD also identified H-bonds formed by the hydroxyl of the carboxyl acid and the nitrogen of the azole ring of ITR (Figure 7 and Table S2), in agreement with the results of vibrational spectroscopy (Figure 6). This interaction was also observed in other cocrystals of ITR [14,35], however, in the ITR–TER cocrystal, another H-bond, of the C–H…O type, was also identified (Figure 7 and Table S2).

As the only single crystal X-ray data for ITR cocrystals available in the Cambridge Structural Database (CSD) are for ITR–succinic acid (ITR–SUC), record REWTUK [33], a more detailed comparison of the two cocrystals was made. Although both cocrystals have their crystal lattice formed by four molecules, the ITR–SUC cocrystal was described as a monoclinic system with a P2_1_/c space group [32].

**Figure 8:**
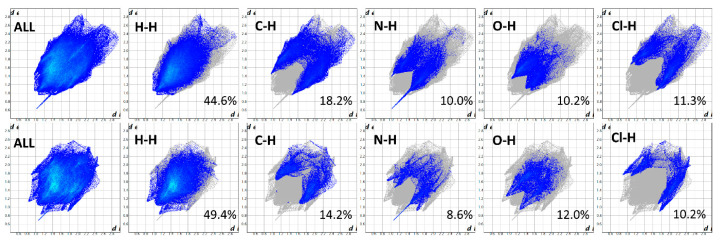
Two-dimensional Hirshfeld fingerprints for ITR–TER (top) and ITR–SUC (bottom) cocrystals.

The 2D fingerprint plots, based on the 3D Hirshfeld surfaces of the ITR molecules in ITR–TER and ITR–SUC cocrystals, were generated to investigate the differences in interactions (Figure 8). It can be seen that the H…H interactions are dominant and represent the most significant contribution (44.6% and 49.4%, in ITR–TER and ITR–SUC cocrystal, respectively) to the total Hirshfeld surfaces. In the ITR–TER cocrystal, the contribution of the C…H contacts is greater (18.2%) in comparison to the ITR–SUC system (14.2%), most likely due to the additional weak C–H…O H-bond. The dominant N–H…O interaction is visible in both systems, showing as a well-defined “horn” when looking at the N…H interactions only (Figure 8). What can be concluded is that the degree of interactions including C…H, N…H and O..H contacts in the ITR–TER systems is higher than that in the TER–SUC cocrystal.

### 3.5. Morphological Analysis

**Figure 9:**
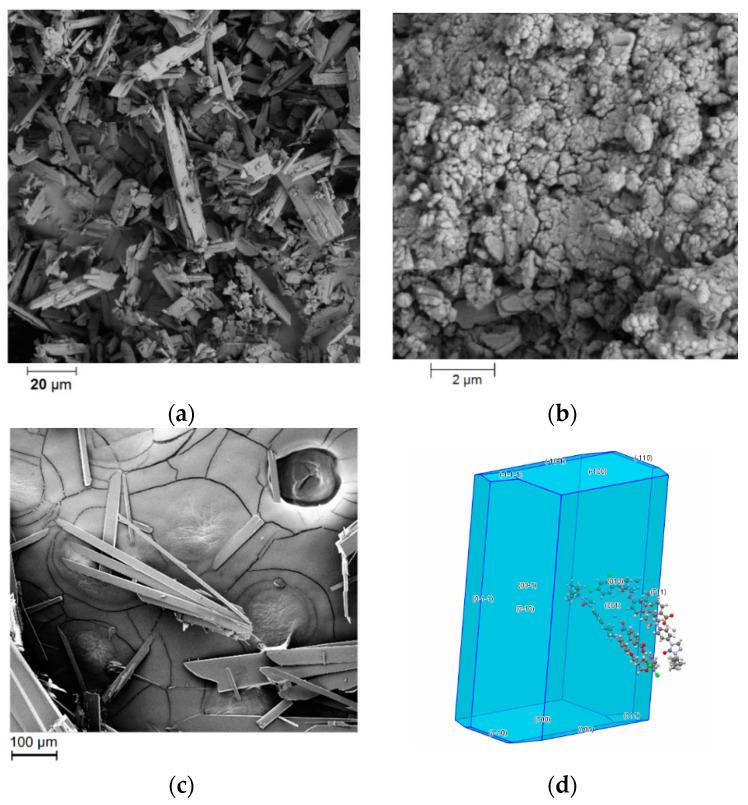
Scanning electron micrographs of: (**a**) crystalline ITR and the ITR–TER cocrystal produced by (**b**) ball milling, (**c**) slow solvent evaporation from methanol, as well as the (**d**) Bravais, Friedel, Donnay and Harker (BFDH) predicted morphology based on the single crystal structure of the ITR–TER cocrystal.

The morphological analysis revealed that the ITR powder “as received” was composed of large rod-like structures, with particles up to 30 µm (Figure 9a). An SE micrograph of particles of ITR–TER cocrystal produced by ball milling showed that this sample was composed of clusters of round particles smaller than 1 µm (Figure 9b), while the cocrystals prepared by slow solvent evaporation from methanol showed larger rod-like structures of approximately 450 µm (Figure 9c). The Bravais, Friedel, Donnay and Harker (BFDH) predicted morphology of the particle, based on the single crystal structure of ITR–TER cocrystal, showed some similarity between the experimental (Figure 9c) and simulated crystal shapes, however the (001) and (00–1) faces are clearly dominant in the former due to the solvent’s impact on the crystal growth (Figure 9d).

### 3.6. Pharmaceutical Evaluation of ITR Cocrystals

Following the identification, solid-state and structural characterization of the new ITR–TER cocrystal, this phase, in terms of its functional, pharmaceutical properties, was compared this those of already described cocrystals: ITR–succinic acid (ITR–SUC, [14]) and ITR–oxalic acid (ITR–OXA, [15]) as well as a freeze dried, disordered form of ITR (ITR FD). In this work, ITR–SUC and ITR–OXA cocrystals were synthesized by ball milling, so as to exclude the impact of the preparative method, and were characterized by PXRD and DSC (Figures S1 and S2) as well as SEM (Figure S3). The PXRD and DSC data for ITR FD are shown in Figure S4, while the scanning electron micrograph is shown in Figure S3.

#### 3.6.1. Contact Angle Analysis

**Figure 10:**
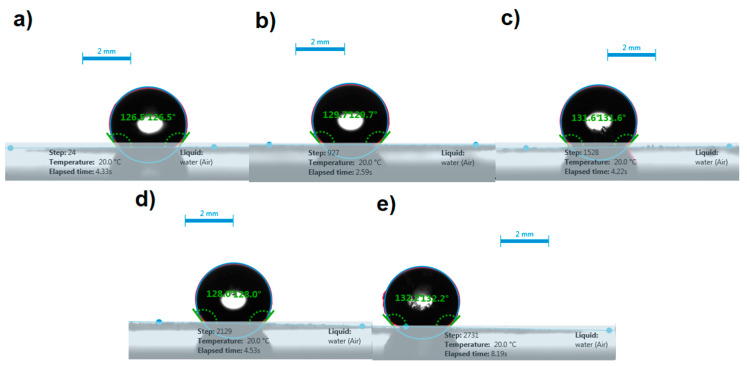
Representative images of water droplets on the surface of disks and contact angles of: (**a**) crystalline ITR, (**b**) freeze dried ITR (FD ITR), (**c**) the ITR–OXA cocrystal, (**d**), the ITR–SUC cocrystal and (**e**) the ITR–TER cocrystal.

The contact angle of the water droplets on the surface of disks prepared by compacting ITR, FD ITR, as well as the ITR–OXA, ITR–SUC and ITR–TER cocrystals, was measured to estimate the wettability of these samples. The photos taken during this experiment showed that the shape of the water droplets remained spherical on the surface of all the disks (Figure 10), highlighting the hydrophobicity of these materials.

The contact angle values of the samples were similar (Table 2), with ITR–SUC and ITR–TER showing the lowest and the highest values, respectively. The values did not change significantly after 10 min of evaluation, in comparison to the initial values, indicating that the prolonged contact of the disks with water droplets had no effect on their wettability. These results indicated that the samples investigated, regardless of the solid-state form, had poor wettability, as the values of the contact angle were greater than 90° [36].

#### 3.6.2. Intrinsic Dissolution Rate (IDR)

**Figure 11:**
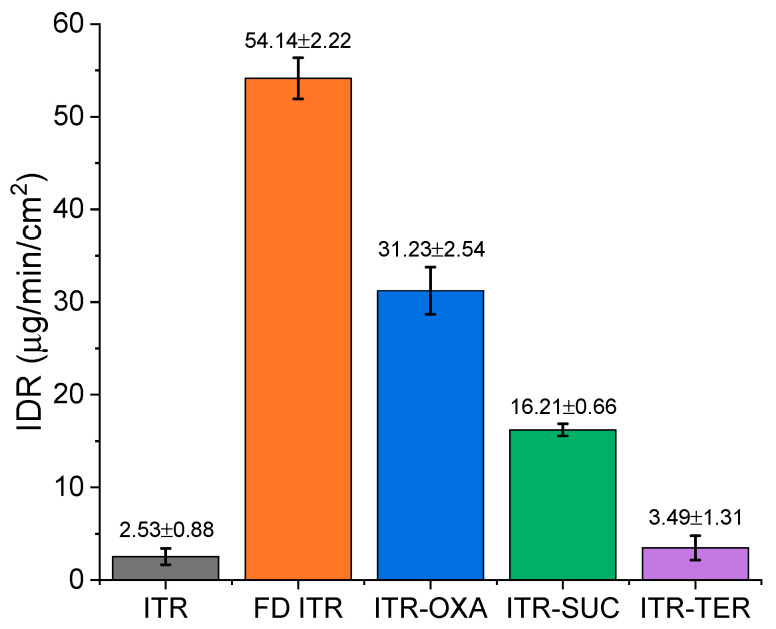
Intrinsic dissolution rates (IDRs) of: ITR starting material powder (ITR), freeze dried ITR (FD ITR), the ITR–OXA cocrystal, ITR–SUC cocrystal and ITR–TER cocrystal.

IDR analysis at pH 1.2 (Figure 11) revealed a different behavior of the samples, with crystalline ITR showing the slowest IDR in comparison to FD ITR, which achieved the highest dissolution rate of all the samples analyzed. The faster IDR of the FD ITR in relation to ITR was related to the disordered state of the API in the former. As identified by the solid-state characterization (Figure S4), the API in FD ITR was in the liquid crystal mesophase, a higher energy form than the crystalline state of ITR (the starting material). Therefore, the high free energy of FD ITR promoted the quicker dissolution of the API [10,37]. A one-way ANOVA revealed that the investigated samples had statistically different IDR values, except for crystalline ITR and the ITR–TER cocrystal. In this case, cocrystallisation was unable to improve the dissolution rate of the API, likely due to the poor aqueous solubility of the coformer, TER, which has an aqueous solubility of 17 µg/mL at 25 °C [38]. This value is low, but still much higher than ITR’s solubility in water of around 1 ng/mL [39]. The influence of the coformer on the IDR values was evident for the other cocrystals, as the rank order of the aqueous solubility of pure coformers was OXA > SUC > TER (OXA: 130–140 mg/mL [40] and SUC: 83 mg/mL [41]), following the rank order of the IDR values of the equivalent cocrystals: ITR–OXA > ITR–SUC > ITR–TER (Figure 11). Raman spectroscopy of the disks post IDR studies showed the conservation of their solid-state characteristics.

#### 3.6.3. Powder/Formulation Dissolution

A powder dissolution analysis was carried out in two stages: one with the samples physically mixed with lactose monohydrate in a 1:6 *w*/*w* API:excipient ratio to improve the wettability and dispersibility of the drug powders, and another with the samples physically mixed with the same excipients as those in the Sporanox^®^ formulation. In both dissolution experiments, Sporanox^®^ capsules, a marketed formulation, was used as the reference. For the purpose of dissolution studies, the pellets were removed from the capsule shells and used without further alteration. While dissolution studies performed on ITR cocrystal particles dominate the literature [14,16], scarce information is available on the behavior during formulation, therefore the inclusion of a range of pharmaceutical excipients in the dissolution studies allows the cocrystal performance to be evaluated better.

**Figure 12:**
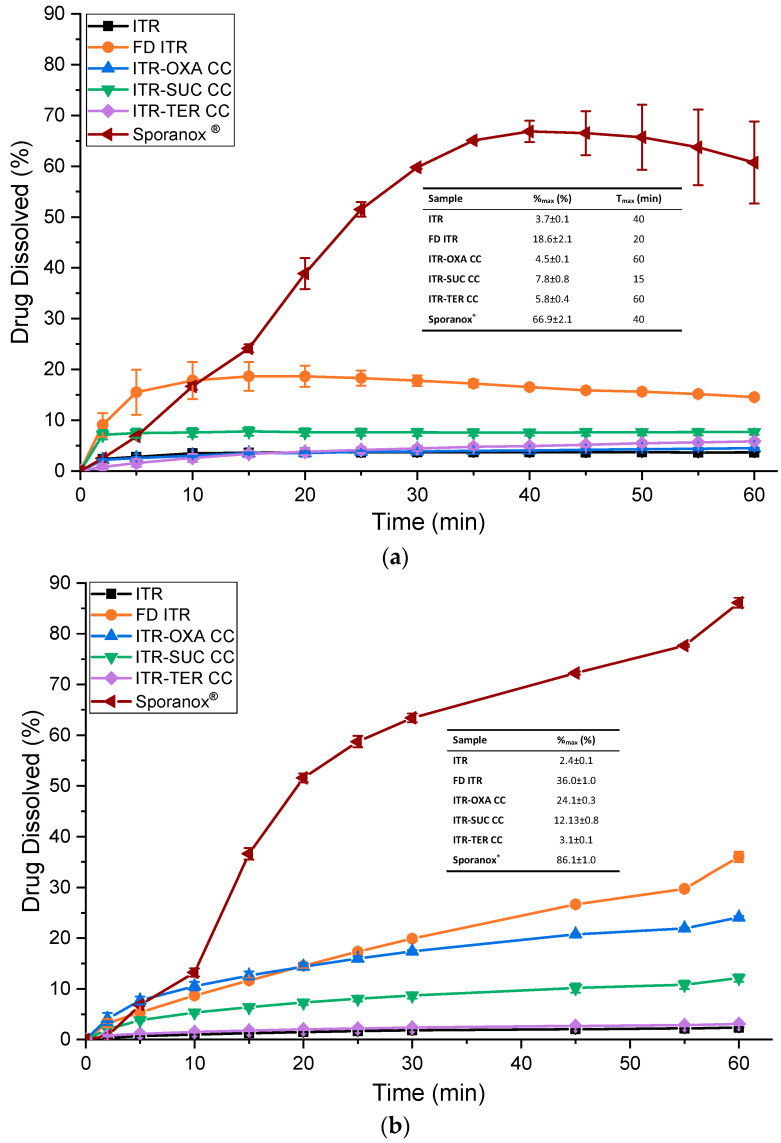
(**a**) Dissolution profile of Sporanox^®^ and the physical mixtures of ITR and cocrystals with lactose monohydrate in a 1:6 ratio (*w*/*w*) of the active pharmaceutical ingredient (API) and excipient; (**b**) dissolution profile of Sporanox^®^ and the ITR, FD IDR, ITR–OXA, ITR–SUC and ITR–TER mixed with excipients present in the Sporanox^®^ capsule. T_max_—the time to reach the maximum percent (%_max_) of the dissolved drug, CC—cocrystal.

In the dissolution experiment where the samples were mixed with lactose, the results showed that Sporanox^®^ had a superior dissolution profile in comparison to the other samples (Figure 12a). ITR from Sporanox^®^ had an almost constant dissolution rate until it reached its maximum percent of drug dissolved, %_max_, of 66.9 ± 2.1% after 40 min. In comparison to ITR, which had a %_max_ of 3.7% ± 0.1%, the concentration of solubilized ITR released from Sporanox^®^ was greater by 18-fold, highlighting the enhanced dissolution performance of this commercial form.

FD ITR and ITR–SUC had faster initial dissolution rates in comparison to other ITR forms (Figure 12a). The time required to reach the maximum percent of dissolved drug, T_max_, was 20 min for FD ITR and 15 min for ITR–SUC, while for the other samples this time was between 40 and 60 min. This effect could be due to the differences in solubility between the various forms. Nevertheless, the dissolution profile of FD ITR showed a decrease in the amount of the dissolved material from 18.6 ± 2.8% to 14.6 ± 0.4% after 60 min, which is statistically different (*t*-test, *p*-value = 0.03). This slight “parachute effect” might be caused by the crystallization of the dissolved API in order to reduce the chemical potential of the supersaturation generated when the disordered API was dissolved [42]. This was confirmed using PXRD, and it was apparent that the material remaining after the dissolution of FD ITR crystallized to form I of ITR [27].

Among the cocrystals, ITR–SUC had the highest %_max,_ of 7.8% ± 0.8%, while the dissolution profile of ITR–OXA mostly overlapped with that of ITR. The cocrystal had a slightly higher %_max_, 4.5% ± 0.1%, while ITR had a %_max_ of 3.7% ± 0.1% (Figure 12a). This result contrasts with the IDR study (Figure 11), which showed a faster dissolution rate of ITR–OXA in relation to ITR. In this study, the very small difference in the percentage of the solubilized drug between ITR and ITR–OXA could be caused by an incongruent dissolution from the cocrystal and the removal of the coformer from its surface. This is due to the very different solubilities of OXA (130–140 mg/mL) in comparison to that of ITR [39,40]. A conversion to crystalline ITR form I was observed by a PXRD analysis of the remaining ITR–OXA. Therefore, the cocrystallisation of an API with a coformer with a very different aqueous solubility might not be able to enhance the dissolution rate of the API [43].

Dissolution studies of the ITR–TER cocrystal showed that the amount of the drug dissolved was increasing until the final measurement at 60 min (Figure 12a), reaching a %_max_ of 5.8% ± 0.4%, which is statistically greater by a *t*-test (*p* = 0.0009) comparison than that of crystalline ITR. The PXRD trace of the undissolved ITR–TER after the dissolution test showed no alteration in the solid-state properties after the test, suggesting that this cocrystal has an enhanced stability in aqueous media in relation to the other samples.

The dissolution analysis using the samples mixed with the same excipients as those present in the Sporanox^®^ formulation (Figure 12b) yielded different results in comparison to the test using the physical mixtures with lactose (Figure 12a). In this experiment, Sporanox^®^ also had a superior dissolution profile when compared to the other samples, reaching 86.1% ± 1.0% of dissolved drug. Only 2.4% ± 0.1% dissolved from crystalline ITR. FD ITR had the second highest dissolution profile. This sample continuously released ITR, reaching a %_max_ of 36.0% ± 1.0%. No “parachute” effect was observed, in contrast to the previous analysis (Figure 12a). This could be caused by the stabilizer effect of the polymers PEG 6 kDa and HPMC, preventing the crystallization of the solubilized API [44]. The PXRD trace of the undissolved FD ITR recovered after the dissolution test showed no alteration of its solid-state characteristics, suggesting that the FD ITR had an enhanced physical stability in this experiment.

In relation to the cocrystals, ITR–OXA reached the greatest %_max_ of 24.1 ± 0.3% (Figure 12b). The ITR–SUC cocrystal had a %_max_ of 12.1 ± 0.8%, while ITR–TER had the lowest but was nevertheless greater than crystalline ITR, with a drug release of 3.1 ± 0.1%. In comparison to the previous analysis, ITR–OXA and ITR–SUC showed improved dissolution profiles, probably due to the presence of polymers in the dissolution media, as observed for the FD ITR sample. The PXRD analysis of ITR–OXA and ITR–SUC after the dissolution test showed no phase change when compared to the samples before dissolution.

## 4. Conclusions

A new itraconazole cocrystal was produced using terephthalic acid, which is the first aromatic coformer described as being able to cocrystallise with ITR. The elucidation of the crystalline structure of the ITR–TER cocrystal revealed that this new form is similar to other known ITR cocrystals with a 2:1 stoichiometry, where the ITR molecules are in an antiparallel arrangement and the coformer is “trapped” in the space formed between the two ITR molecules. However, this supramolecular arrangement had a different heterosynthon in comparison to the existing cocrystals based on aliphatic dicarboxylic acids with an additional H-bond stabilizing the structure.

A comparison of the dissolution behavior of the various forms of ITR, including crystalline ITR, freeze dried ITR and ITR cocrystals with oxalic acid, succinic acid and terephthalic acid, showed that they had different dissolution characteristics. FD ITR showed a remarkable improvement in terms of its dissolution, considering IDR and the powder dissolution in relation to crystalline ITR. This was due to the high energy state of the API achieved by freeze drying. For the cocrystals, their IDR values and powder dissolution profiles correlated with the aqueous solubility of the coformers. The rank order of the dissolution rates of the API from the cocrystals was ITR–OXA > ITR–SUC > ITR–TER. Furthermore, physically mixing FD ITR, ITR–OXA and ITR–SUC with the same excipients as those present in the Sporanox^®^ formulation enhanced the stability of these forms in the dissolution media, preventing their transformation into the crystalline parent API.

## Figures and Tables

**Table 1:** Composition of the powders used in the dissolution study.

Excipient (%, *w*/*w*)	Formulation				
	ITR	FD ITR	ITR–OXA	ITR–SUC	ITR–TER
API	21.74	21.74			
Cocrystal			23.22	23.13	23.70
Sucrose	41.74	41.74	40.95	41.0	40.69
HPMC ^(^*^)^	32.61	32.61	31.99	32.03	31.79
PEG ^(^**^)^	3.91	3.91	3.84	3.84	3.82
Total (%)	100	100	100	100	100
Powder weight (mg) ^(^***^)^	460	460	501.1	498.32	514.2

(*) Hydroxypropyl methyl cellulose 2910 (5 mPa.s (HPMC)); (**) polyethylene glycol 6000 Da (PEG); (***) mass of the powder containing 100 mg of ITR used in the dissolution test.

**Table 2:** Contact angle of a water droplet at the start and end of the measurement as well as the statistical analysis indicating no difference between the initial and final values.

Sample	Initial (°)	Final * (°)	*p*-Value
ITR	126.8 ± 0.4	126.5 ±0.3	0.27
FD ITR	128.9 ± 0.8	127.0 ± 1.7	0.16
ITR–OXA	128.7 ± 2.9	126.0 ± 3.5	0.35
ITR–SUC	126.5 ± 1.5	124.8 ± 2.0	0.30
ITR–TER	130.5 ± 1.7	129.7 ± 1.9	0.61

^(^*^)^ After 10 min of droplet deposition.

## Supplementary Materials

The following are available online at https://www.mdpi.com/1999-4923/12/8/741/s1, Figure S1: (**a**) PXRD patterns of itraconazole (ITR) and oxalic acid (OXA) starting material powders and the ITR–OXA cocrystal produced by ball milling (BM). ***** Indicates distinct peaks of the cocrystal. (**b**) DSC thermograms of ITR and OXA starting material powders and the ITR–OXA cocrystal, Figure S2: (**a**) PXRD patterns of the itraconazole (ITR) and succinic acid (SUC) starting material powders and the ITR–SUC cocrystal produced by ball milling (BM). ***** Indicates distinct peaks of the cocrystal. (**b**) DSC thermograms of the ITR and SUC starting material powders and the ITR–SUC cocrystal, Figure S3: Scanning electron micrographs of (**a**) the ITR–SUC cocrystal and (**b**) ITR–OXA cocrystal produced by ball milling; (**c**) freeze dried ITR, Figure S4: (**a**) PXRD pattern of freeze dried itraconazole (ITR FD) and (**b**) DSC thermogram of freeze dried itraconazole (ITR FD), Table S1: Crystal data and structure refinement for the ITR–TER cocrystal, Table S2: Hydrogen bonds for ITR–TER cocrystal [Å and °]. Crystallographic data, deposition number 2015894, were deposited with the Cambridge Crystallographic Data Centre. These data can be obtained free of charge from the Cambridge Crystallographic Data Centre via www.ccdc.cam.ac.uk/structures.
