# Supplementary material for: Identification and Pharmaceutical Characterization of a New Itraconazole Terephthalic Acid Cocrystal
Source: Pharmaceutics. 2020 Aug 6;12(8):741. doi: 10.3390/pharmaceutics12080741 (PMC7464664; doi:10.3390/pharmaceutics12080741)
Supplement: Supplementary file 1 [file pharmaceutics-12-00741-s001.pdf]

pharmaceutics

Supplementary Materials: Identification and Pharmaceutical Characterization of a New Itraconazole Terephthalic Acid Cocrystal

Ricardo Machado Cruz, Tereza Boleslavská, Josef Beránek, Eszter Tieger, Brendan Twamley, Maria Jose Santos-Martinez, Ondřej Dammer and Lidia Tajber

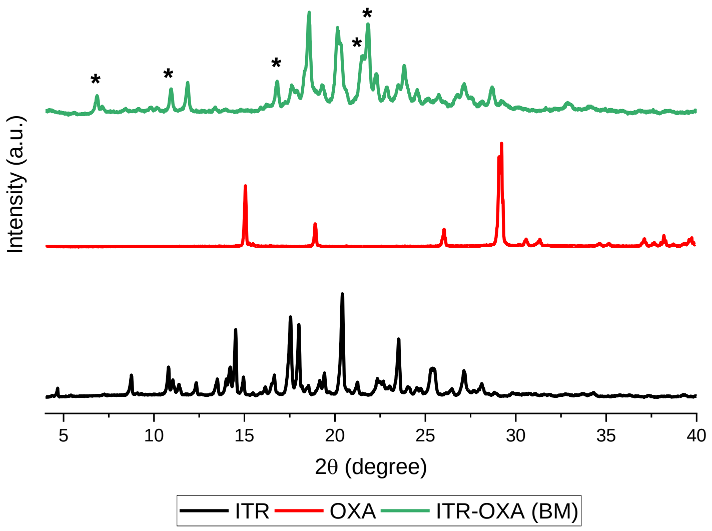

(a)

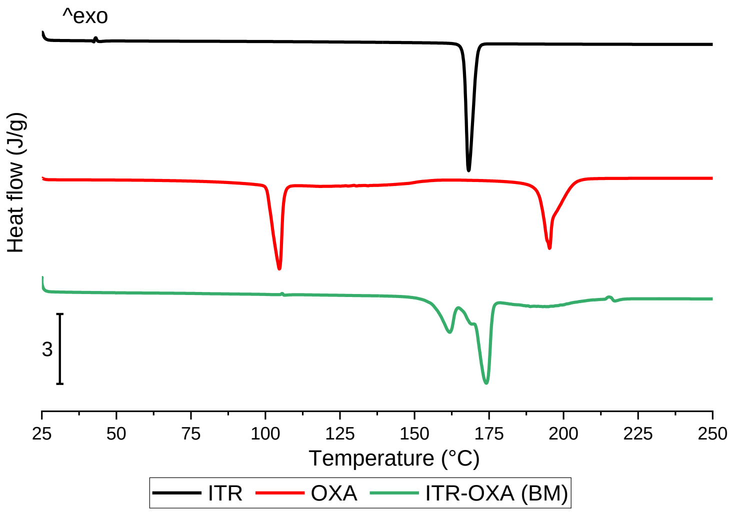

(b)

Figure S1. (**a**) PXRD patterns of itraconazole (ITR) and oxalic acid (OXA) starting material powders and ITR-OXA cocrystal produced by ball milling (BM). * indicate distinct peaks of the cocrystal. (**b**) DSC thermograms of ITR and OXA starting material powders and ITR-OXA cocrystal.

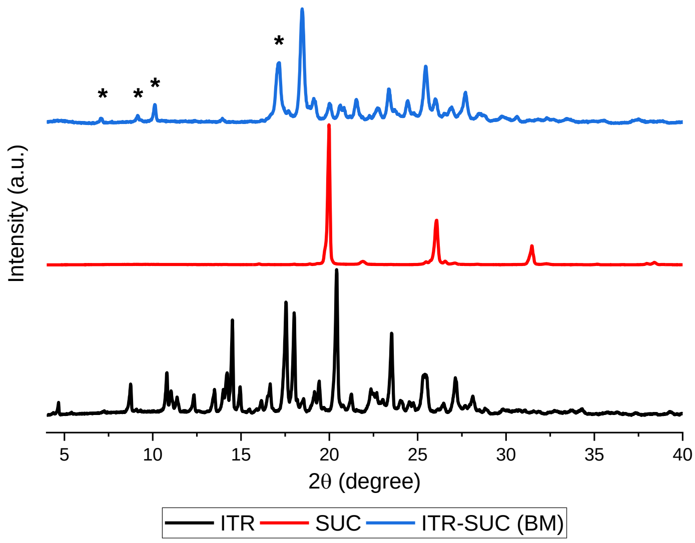

(a)

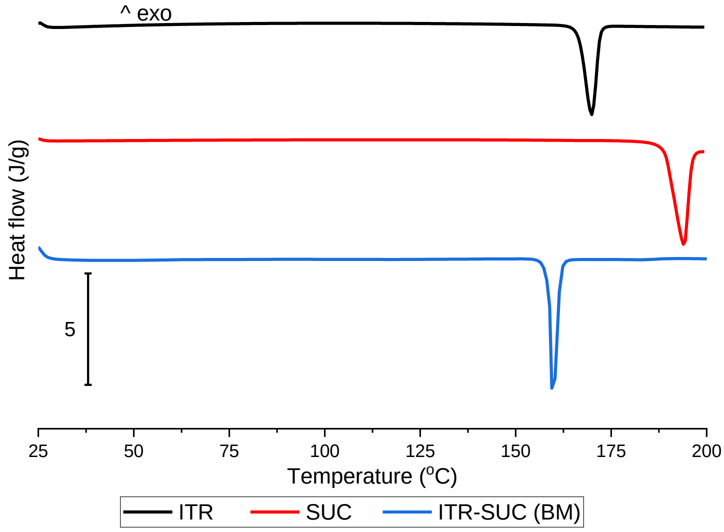

Figure S2. (**a**) PXRD patterns of itraconazole (ITR) and succinic acid (SUC) starting material powders and ITR-SUC cocrystal produced by ball milling (BM). * indicate distinct peaks of the cocrystal. (**b**) DSC thermograms of ITR and SUC starting material powders and ITR-SUC cocrystal.

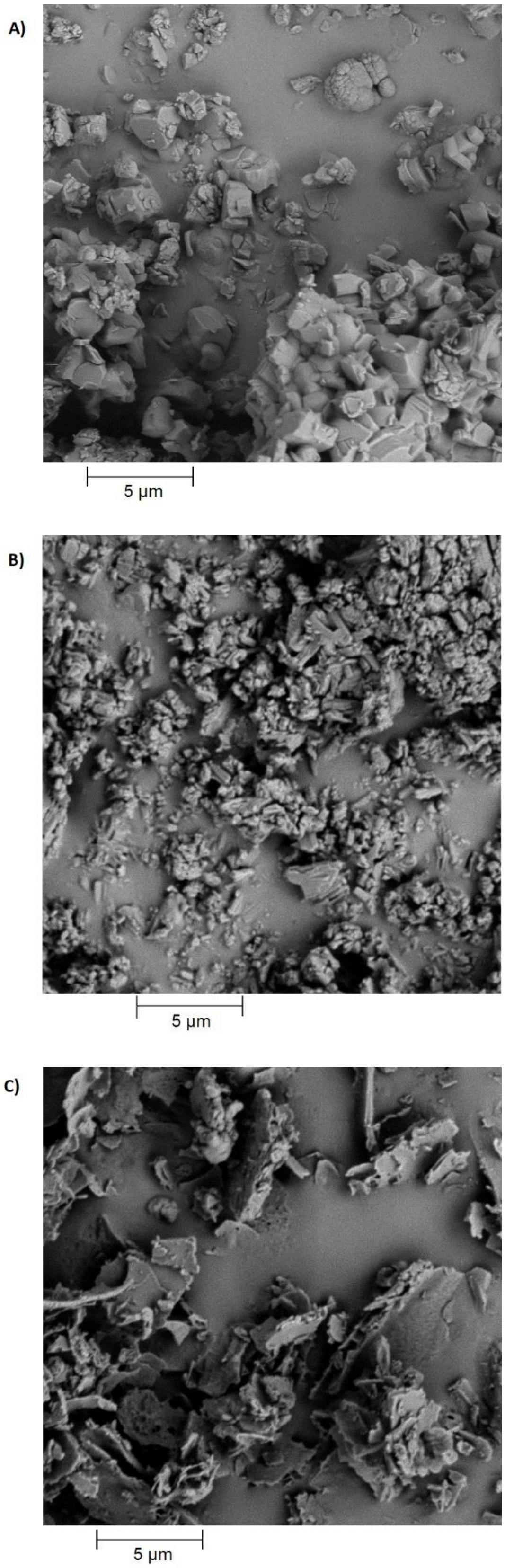

Figure S3. Scanning electron micrographs of (**a**) ITR-SUC cocrystal and (**b**) ITR-OXA cocrystal produced by ball mill; (**c**) freeze dried ITR.

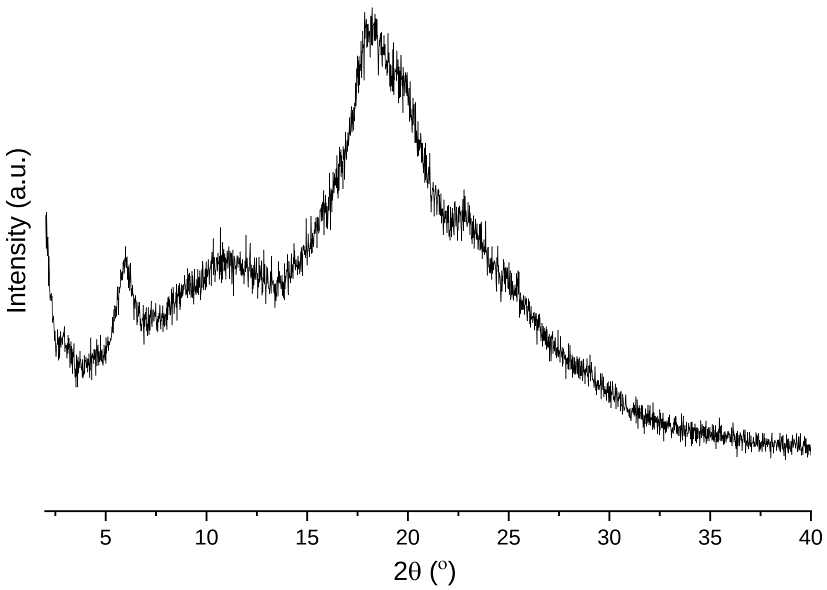

(a)

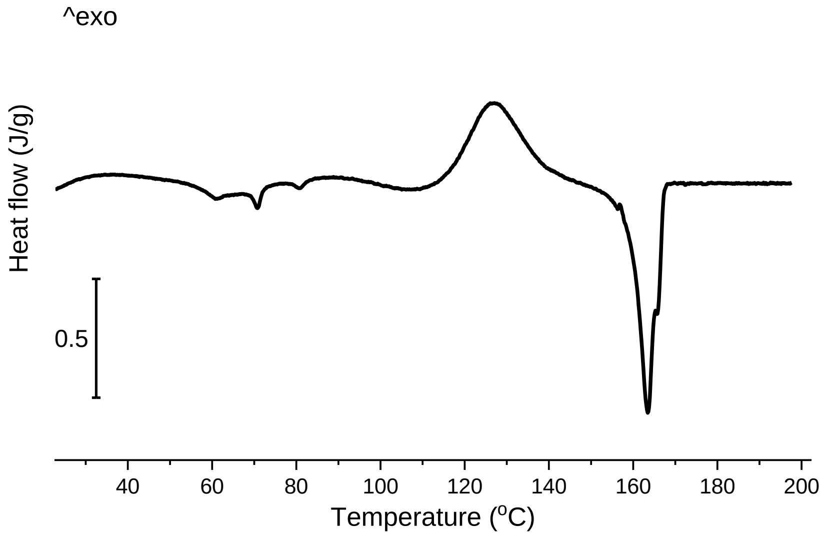

(b)

Figure S4. (**a**) PXRD pattern of freeze dried itraconazole (ITR FD) (**b**) DSC thermogram of of freeze dried itraconazole (ITR FD).

Table S1. Crystal data and structure refinement for ITR-TER cocrystal.

CCDC No.	2015894	
Empirical formula	$C_{39}H_{41}Cl_2N_8O_6$	
Formula weight (Da)	788.70	
Temperature of measurement (K)	100 ± 2	
Wavelength (Å)	1.54178	
Crystal system	Triclinic	
Space group	$P\bar{1}$	
Unit cell dimensions (Å, °)	a = 10.3910±6	α = 81.310 ± 5
	b = 14.3714 ± 10	β = 81.019 ± 6
	c = 25.418 ± 2	γ = 89.951 ± 4
Volume (Å^3)	3705.2 ± 5	
Z	4	
Calculated density (mg/m^3)	1.414	
Absorption coefficient (mm^{-1})	2.075	
F(000)	1652	
Crystal size (mm^3)	0.15 × 0.14 × 0.03	
Theta range for data collection (°)	1.780 to 66.203	
Index ranges	−12≤h≤12, −15≤k≤16, −29≤l≤14	
Reflections collected	12852	
Independent reflections	12852 [R(int) = 0.1182]	
Completeness to theta = 66.203°	97.5 %	
Absorption correction	Semi-empirical from equivalents	
Max. and min. transmission	0.752586 and 0.547278	
Refinement method	Full-matrix least-squares on F^2	
Data / restraints / parameters	12852 / 435 / 1076	
Goodness-of-fit on F^2	1.093	
Final R indices [I>2σ (I)]	$R1$ = 0.1069, w$R2$ = 0.2616	
R indices (all data)	$R1$ = 0.1188, w$R2$ = 0.2689	
Largest diff. peak and hole	0.703 and -1.004 e.Å^{-3}	

Table S2. Hydrogen bonds for ITR-TER cocrystal [Å and °].

D-H...A	d(D-H)	d(H...A)	d(D...A)	<(DHA)
C(10)-H(10)...O(50)	0.95	2.53	3.144(11)	123
C(63)-H(63)...O(103)	0.95	2.48	3.122(11)	125
O(48)-H(48)...N(9)	0.84	1.81	2.645(10)	171
O(101)-H(101)...N(62)	0.84	1.81	2.632(10)	167
